# A Variable Cross-Section Microfluidic Channel for Simultaneous Reproduction of Low Oscillatory and Pulsatile Wall Shear Stress at the Carotid Bifurcation: A Computational Fluid Dynamics-Based Study

**DOI:** 10.3390/bios15100648

**Published:** 2025-09-30

**Authors:** Yong-Jiang Li, Hui-Min Hou, Qi-Fei Hu, Li-Jin Yuan, Chun-Dong Xue, Dong Chen, Xu-Qu Hu, Kai-Rong Qin

**Affiliations:** 1 Institute of Cardio-Cerebrovascular Medicine, Central Hospital of Dalian University of Technology, Dalian 116033, China; yongjiangli@dlut.edu.cn (Y.-J.L.); huqifei2024@mail.dlut.edu.cn (Q.-F.H.); xuechundong@dlut.edu.cn (C.-D.X.); huxuqu@dlut.edu.cn (X.-Q.H.); 2 School of Biomedical Engineering, Faculty of Medicine, Dalian University of Technology, Dalian 116024, China; 3 School of Optoelectronic Engineering and Instrumentation Science, Dalian University of Technology, Dalian 116024, China; houhuimin@mail.dlut.edu.cn (H.-M.H.); ylj1@mail.dlut.edu.cn (L.-J.Y.)

**Keywords:** microfluidic channel, pulsatile wall shear stress, oscillatory wall shear stress, carotid bifurcation, computational fluid dynamics

## Abstract

Pulsatile blood flow generates complex wall shear stress (WSS) patterns at the carotid bifurcation, which critically regulate endothelial function and structure. While physiological pulsatile WSS (PWSS) is essential for maintaining vascular health, low oscillatory WSS (OWSS) near the carotid sinus is closely associated with endothelial dysfunction, atherosclerotic plaque formation, and stenosis. Reproducing these hemodynamic conditions in vitro is therefore crucial for investigating endothelial mechanobiology and elucidating the pathogenesis of atherosclerosis. Although microfluidic technologies have emerged as promising platforms for simulating either pulsatile or oscillatory WSS, a system capable of simultaneously replicating both characteristic waveforms—as found in vivo at the carotid bifurcation—remains undeveloped. In this study, we designed a variable cross-section microfluidic channel using Computational Fluid Dynamics (CFD) simulations. Numerical results demonstrate that the optimized channel accurately reproduces low OWSS at a stepped section emulating the carotid sinus, alongside high PWSS in a downstream uniform section. Vortex formation induced by the step structure is identified as key to generating low OWSS, influenced by step height, channel width ratio, and input flow rate. This work provides a novel and robust methodology for designing microfluidic systems that mimic complex hemodynamic microenvironments, facilitating future studies on the interplay between distinct WSS patterns and endothelial dysfunction.

## 1. Introduction

The carotid bifurcation is a crucial region in the circulatory system where the common carotid artery splits into the internal and external carotid arteries. The carotid sinus, located at the beginning of the internal carotid artery, is characterized by a slightly thinner vascular wall and an expanded lumen. This anatomical region is particularly vulnerable to the development of atherosclerotic plaques, which can lead to serious cardiovascular events, including carotid artery stenosis and stroke [1,2,3,4,5,6,7,8,9]. One of the key drivers of plaque formation is the hemodynamic environment, especially wall shear stress (WSS), which regulates endothelial cell behavior and modulates localized disease processes. At the carotid bifurcation, shear stress exhibits complex oscillatory flow patterns, rendering this region critically important for studying the initiation and progression of atherosclerosis.

Wall shear stress is a hemodynamic parameter. At carotid bifurcation and near the carotid sinus, WSSs are typically categorized into two regimes: low oscillatory wall shear stress (OWSS) and normal pulsatile shear stress (PWSS). Extensive research [1,2,3,4,5,6,7,8,9] indicates that low OWSS is often associated with regions prone to atherosclerotic plaque formation, whereas NWSS is generally observed in healthy arterial vascular regions. Hence, understanding the impact of these distinct shear stress patterns on vascular endothelial dysfunction provides insight for the mechanobiological mechanisms of atherosclerosis development and progression [10,11,12].

Animal models and human clinical trials provide direct insight for mechanobiological studies. However, they are affected by physiological factors such as respiration and neural activity, and are also hindered by individual variability, high costs, long observation periods, and ethical concerns. In contrast, in vitro models that simulate different WSS waveforms provide a more controlled environment for quantitatively exploring the relationship between WSS and endothelial functions, along with their underlying mechanobiological mechanisms [13]. A parallel-plate flow chamber was the first widely used in vitro model for simulating fluid shear stress. Subsequently, some researchers [14] proposed a parallel-plate flow chamber with a stepped structure to recreate OWSS for mechanobiological studies [10,15,16,17,18,19]. Despite their utility, these systems for simulating oscillatory WSS typically suffer from limitations such as large size and complex external control equipments.

Recent advances in microfluidic technology have facilitated the creation of highly controlled and reproducible hemodynamic environments, enabling quantitative studies of various WSS patterns on vascular endothelial functions [20,21,22,23]. These microfluidic systems provide a powerful tool for investigating endothelial cell behavior under complex, dynamic WSS profiles observed in vivo [24,25,26,27]. However, most existing in vitro models offer a qualitative and generalized representation of oscillatory WSS, failing to accurately replicate the intricate characteristics of WSS, particularly those associated with carotid bifurcation and the carotid sinus. Moreover, there remains a gap in the ability to simultaneously replicate both low OWSS and PWSS at carotid bifurcation within a single microfluidic platform. Computational Fluid Dynamics (CFD) is widely used to model physiological blood flow and to design and validate microfluidic chips [28,29]. Some studies [30,31] numerically investigated the effect of bifurcation geometry, internal carotid artery stenosis, and recanalization therapy on hemodynamic variables in the carotid artery. Our previous work [13,32] designed microfluidic devices for precisely reproducing physiological and exercise-induced WSS. Nevertheless, a microfluidic channel capable of simultaneously replicating both low oscillatory WSS (associated with carotid sinus stenosis) and normal pulsatile WSS (observed in healthy arterial segments) at the carotid bifurcation has not been previously achieved in a single platform.

This study presents the design of a variable cross-section microfluidic channel capable of simultaneously reproducing low-OWSS and high-PWSS patterns, which are representative WSSs of the carotid sinus and uniform arterial segments at carotid bifurcation. Using a real carotid artery stenosis model, low OWSS and high PWSS were obtained with CFD analysis and served as a criterion for the design of the microfluidic channel. A variable cross-section microfluidic channel with stepped structure is developed. To simultaneously reproduce the targeted WSS patterns, the influence of microfluidic channel geometry and flow rates is systematically characterized and optimized.

## 2. Materials and Methods

### 2.1. Extraction of WSS Patterns at the Carotid Bifurcation

**Figure 1:**
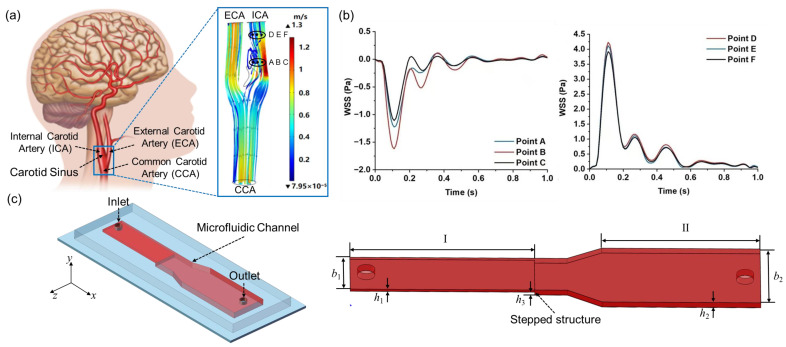
(**a**) Anatomy of the human common carotid artery (CCA), external carotid artery (ECA), internal carotid artery (ICA), and carotid sinus, along with the velocity distribution at peak systole; (**b**) low-OWSS waveforms at the site of carotid sinus stenosis and high-PWSS waveforms in the downstream segment of the ICA; (**c**) schematic diagram and geometric structure of the variable cross-section microfluidic channel.

To capture typical WSS patterns at the carotid sinus and downstream arterial segments of the carotid bifurcation, numerical simulations of the flow field were conducted (Figure 1a). A three-dimensional (3D) carotid bifurcation model, reconstructed from human medical imaging data, included the common carotid artery (CCA), the internal carotid artery (ICA) with an enlarged carotid sinus, and the external carotid artery (ECA) [33]. A protruding plaque at the carotid sinus was integrated into the model to simulate in vivo stenosis conditions. The total model length is 52 mm, with diameters of 6.4 mm for the CCA, 2.6 mm for the ICA, and 2.2 mm for the ECA. The axial blood flow velocities in the CCA were measured using ultrasound Doppler (see Figure S1), and a uniform velocity profile (constant across the entire inlet cross-section) was applied at the CCA inlet. Zero-pressure boundary conditions were set at the ICA and ECA outlets [30,34]. Based on the low Reynolds number laminar flow and under the assumption of no-slip conditions, the velocity field and WSS distribution at the carotid bifurcation were numerically analyzed. The resulting low OWSS at the disturbed flow site of carotid sinus stenosis and high PWSS in the downstream uniform arterial segments (Figure 1b) were identified as the target WSS waveform features for simulation in the microfluidic channel.

### 2.2. Design of the Variable Cross-Section Microfluidic Channel

To effectively replicate both the low OWSS at the carotid sinus and the high PWSS in the downstream uniform arterial segments, we developed a variable cross-section microfluidic channel with a stepped design, based on principles of fluid dynamics and microfluidic chip technology. As illustrated in Figure 1c, this channel consists of a stepped variable cross-section flow chamber and circular inlet and outlet with radius R. The stepped flow chamber is divided into two main parts: (I) a narrower rectangular cross-section channel with a stepped structure at the front and (II) a wider rectangular cross-section channel at the rear, connected with a transitional cross-section channel. The stepped height transition in part (I) induces vortex formation near its rear edge, effectively simulating the low-OWSS waveform at the carotid sinus [31,35]. In contrast, the wider rear channel (II) exhibits laminar flow (Reynolds number is ∼0.2), making it suitable for modeling the high-PWSS downstream arterial segments of the carotid bifurcation. By adjusting the geometric dimensions of the two parts of the microfluidic channel, such as step height in channel (I) and the width of channel (II), the system can precisely simulate both low-OWSS and high-PWSS patterns of the carotid bifurcation.

### 2.3. Governing Equations of Fluid Motion in the Microchannel

Assuming that the fluid in the microfluidic channel behaves as an incompressible Newtonian fluid, the flow dynamics is governed by the Navier–Stokes equation

(1) $$\frac{\partial \vec{V}}{\partial t}+\left(\vec{V}\cdot \nabla \right)\vec{V}=-\frac{1}{\rho }\nabla p+\frac{\mu }{\rho }\nabla^{2}\vec{V},$$

and the continuity equation

(2) $$\nabla \vec{V}=0,$$

where $\vec{V}$ represents the fluid velocity, μ denotes the fluid viscosity, and ρ signifies the liquid density (Table 1).

Assuming a uniform velocity profile at the inlet of the microfluidic channel, the boundary conditions are defined as

(3) $$\vec{V_{i}}=-\frac{Q(t)}{\pi R^{2}}\vec{j},$$

(4) $$\vec{V_{w}}=0,$$

(5) $$p_{0}=0,$$

where $\vec{V_{i}}$ denotes the inlet velocity, *Q*(t) represents the time-dependent volumetric flow rate at the inlet, $\vec{j}$ is the unit vector along the y-axis in the Cartesian coordinate system, $\vec{V_{w}}$ is the velocity at the channel wall, and *p*_0_ is the pressure at the outlet.

After determining the flow field velocity distribution through numerical simulations, the WSS at the bottom of the microfluidic channel downstream of the stepped structure (*x* = 14–17 mm, *y* = −*h*_2_) is calculated as:

(6) $$\tau_{w1}=\mu \frac{\partial u}{\partial y}{|}_{y=-h_{2}}$$

Under quasi-steady flow conditions, the WSS at the bottom of the wider rectangular channel (II) is calculated using the formula [36]:

(7) $$\tau_{w_{2}}\left(t\right)=\frac{6\mu Q(t)}{b_{2}h_{2}^{2}}$$

Here, *Q*(t) is the volumetric flow rate entering the microfluidic channel, while b_2_ and h_2_ represent the width and height, respectively, of the wider rectangular cross-section channel (II).

### 2.4. CFD Simulation and Optimization of WSS in the Microchannel

**Figure 2:**
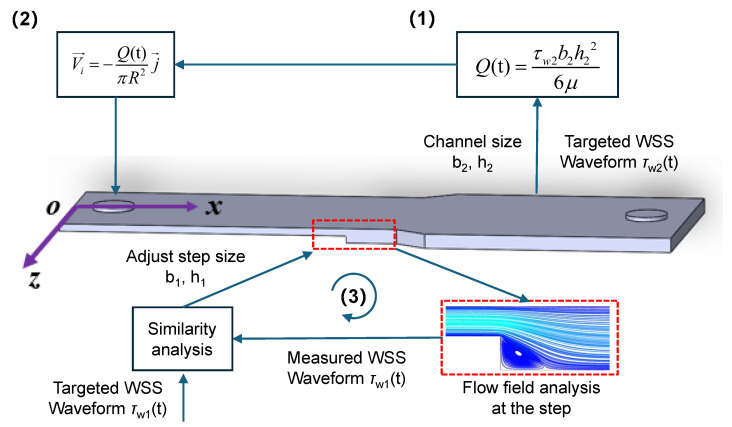
Schematic of the optimization procedures ((1)–(3)) for CFD simulation and geometric optimization of the microfluidic channel.

To replicate in vivo WSS patterns of carotid bifurcation within the microfluidic channel, CFD analysis was conducted for both WSS modeling and microchannel dimensional optimization. As depicted in Figure 2, the process began by calculating the volumetric flow rate *Q*(t) in the microchannel using Equation (7), guided by the high PWSS waveform in the downstream uniform arterial segment (shown in Figure 1b), and under the predefined channel dimensions *h*_2_ = 0.15 mm and *b*_2_ = 5 mm. Subsequently, the corresponding inlet velocity waveform was determined using Equation (3). The step dimensions (*b*_1_ and *h*_3_) were systematically adjusted to minimize the root mean square error (RMSE) between the simulated wall shear stress (WSS) waveforms at five observation points ($x_{1}-x_{5}$) in the vortex region and the target in vivo WSS profile (Table 1). The average RMSE across these points served as the optimization criterion. A threshold of 0.15 was established based on preexperimental analysis, ensuring that the simulated oscillatory WSS (OWSS) waveform closely matched the expected physiological pattern both quantitatively and qualitatively. This value was selected as it represents a margin of error significantly smaller than the physiologically relevant differences in WSS magnitude between distinct hemodynamic regions, thereby guaranteeing that the resulting mechanical environment remains biologically representative. Iterations were performed until a configuration meeting this criterion was identified, confirming the channel’s capability to accurately replicate the target hemodynamic conditions.

In optimizing the step dimensions within the microchannel, the influence of step height h_3_ and width b_1_ on the OWSS waveform was initially analyzed, setting the heights and widths to *h*_3_ = 0.03 mm, 0.06 mm, and 0.09 mm and *b*_1_ = 2 mm, 3 mm, and 4 mm, respectively. The step was subsequently optimized to attain the desired fitting effect. During optimization, the step height varied in increments of 0.03 mm and the width in increments of 1 mm. Furthermore, by altering the width of the rear wider part (II) to *b*_2_ = 4 mm, the impact of *Q*(t) variations on the OWSS waveform at the stepped region was examined following the same optimization protocol. The optimized 3D model was chosen to assess the WSS waveform consistency in both the downstream expanded segment and the stepped region. Using the coordinate system from Figure 2, five points at different x-positions were selected in both areas along the central axis of the microchannel (*z* = 0 mm, *y* = −0.15 mm) to examine the WSS waveform. Additionally, vortex changes at the moments of maximum (t_p_= 0.11 s) and average (t_m_ = 0.16 s) flow velocities were observed at different cross-sectional areas. Lastly, to directly investigate how the low OWSS waveform varied with changes in the cross-sectional area of part (I), the waveform was specifically extracted at the center point of the vortex.

The CFD simulation process began by importing the microchannel model, designed in SolidWorks 2021, into COMSOL 6.0 software for mesh generation. The inlet velocity, determined based on the high PWSS waveform anticipated in the downstream microchannel, was set as the inlet condition. The channel walls were defined as no-slip surfaces, and the outlet was set to zero pressure. Mesh independence verification was performed in a flow chamber with a narrow section width of *b*_1_ = 4 mm and step height of *h*_3_ = 0.06 mm (see Figure S2). By balancing the accuracy and computational efficiency, mesh generation was conducted with the regular–finer mode, which involves selecting the regular option in the mesh generation settings of COMSOL Multiphysics 6.0, with the finer option chosen at the step ranging from 13.5 mm to 14.5 mm. The CFD simulation was conducted by laminar transient solver based on finite element, with a constant time step of 0.01 s.

## 3. Results

Employing a 3D reconstructed carotid artery model from clinical imaging and CFD numerical simulations, we obtained the low OWSS waveform at the carotid sinus and the high PWSS waveform at the uniform downstream section of the carotid bifurcation. Utilizing the design of a variable cross-section microchannel and controlled inlet flow, we systematically analyzed the influences of channel width b_1_, step height h_3_, and input flow rate *Q*(t) on the WSS within the microchannel. Through optimization of the microchannel dimensions, we achieved a more precise simulation of both low OWSS and high PWSS waveforms in different regions of the microchannel.

### 3.1. Carotid Artery WSS Waveform Simulation in the Variable Cross-Section Microchannel

**Figure 3:**
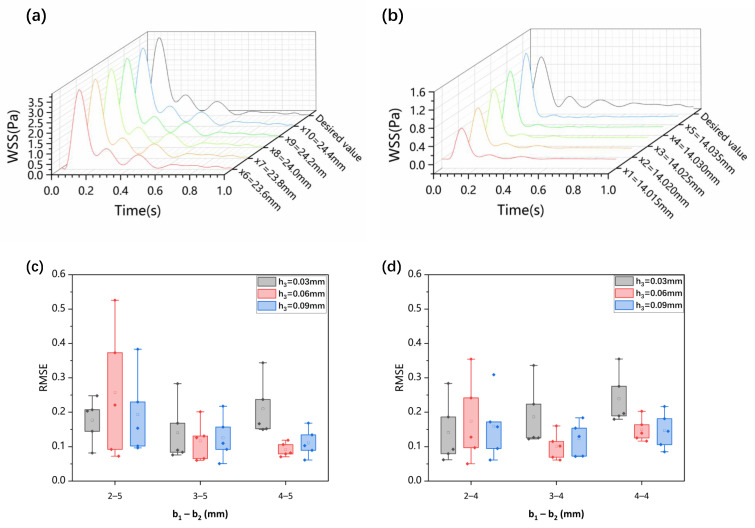
(**a**) WSS waveforms in the wider rectangular region of the microfluidic channel; (**b**) absolute value of low-oscillatory WSS waveforms at the stepped site; (**c**) RMSE between the simulated WSS at five points in the vortex region and desired WSS waveforms with variations in step height h_3_ and width b_1_ at b_2_ = 5 mm; (**d**) RMSE between the simulated WSS at five points in the vortex region and desired WSS waveforms with variations in step height h_3_ and width b_1_ at b_2_ = 4 mm.

Based on the target WSS waveforms at the carotid bifurcation (Figure 1b), and following the CFD simulation and optimization process for the microchannel (Figure 2), we obtained simulation results for WSS in different regions of the optimized microchannel (Figure 3a,b). Within the expanded downstream segment of the microchannel (h_2_ = 0.15 mm, b_2_ = 5 mm), the WSS waveform closely matched the targeted high-PWSS waveform in the internal carotid artery. This consistency demonstrates that the microchannel structure can accurately replicate the high-PWSS waveform in the segment downstream of the carotid bifurcation. Along the fluid flow direction, WSS changes remained consistent, allowing for precise simulation of high pulsatile WSS in this laminar flow region. In the stepped region, we selected five points (x_1–5_) in the vortex region to calculate the RMSE between their WSS and desired WSS. When the geometric parameters were set to b_1_ = 0.04 mm, h_3_ = 0.06 mm, and b_2_ = 5 mm, the RMSE values at these points consistently remained around 0.1, which was in good agreement with expectations (Figure 3c,d). After optimizing the channel dimensions (h_3_ = 0.06 mm, b_1_ = 4 mm), the produced low-OWSS waveform at the step showed similarity to the WSS changes at the carotid sinus stenosis in terms of oscillation pattern and amplitude. The subtle variations in the WSS waveform along the fluid flow direction were attributed to the vortices formed in the stepped area, where differences in velocity gradients at various positions cause WSS variations. Thus, the optimized variable cross-section microchannel successfully simulates both low-OWSS and high-PWSS waveforms of the carotid artery.

### 3.2. Impact of Step Size on Low Oscillatory WSS

**Figure 4:**
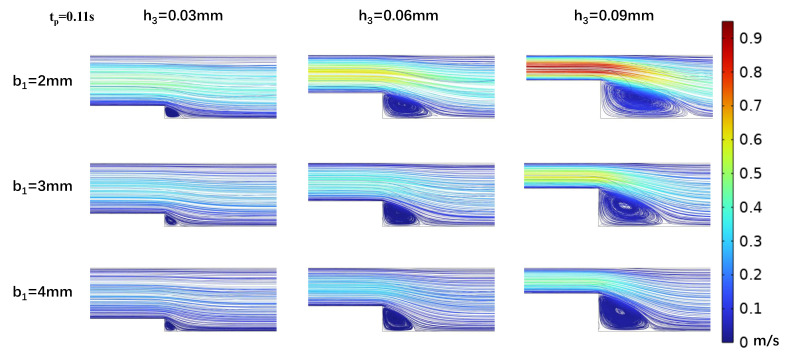
Changes in streamline distributions with step height and width at the stepped site in the time interval of the maximum flow velocity (t_p_ = 0.11 s) for b_2_ = 5 mm.

**Figure 5:**
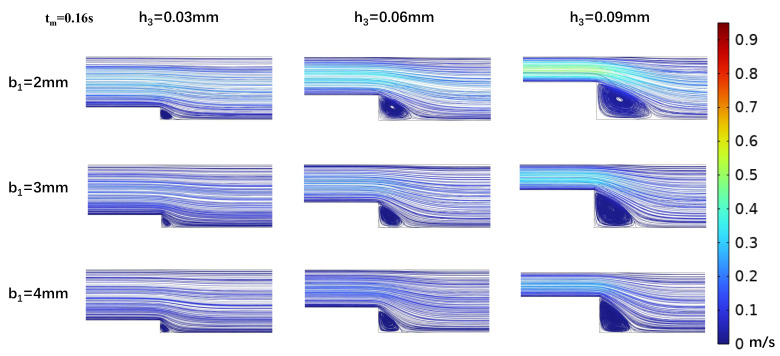
Changes in streamline distributions with step height and width at the stepped site in the time interval of the average flow velocity (t_m_ = 0.16 s) for b_2_ = 5 mm.

In efforts to precisely simulate the low-OWSS waveform, we analyzed the effects of step height h_3_ and width b_1_ on the low OWSS generated in the stepped area. Vortex dynamics were analyzed at peak (t_p_ = 0.11 s) and mean (t_m_ = 0.16 s) flow velocities (Figure 4 and Figure 5), and it was found that as the width of the microchannel step (b_1_) increased, the vortex area in the oxy plane gradually decreased. At h_3_ = 0.03 mm, the vortex area even tended to disappear with b_1_ increasing, making low OWSS simulation challenging. The size of the vortex area is inversely proportional to the change in the step width b_1_ and directly proportional to the flow rate at different moments in the cardiac cycle. As the flow rate decreased, the size of the vortex in the stepped area also decreased. When the microchannel dimensions were b_1_ = 2 mm and h_3_ = 0.03 mm, the vortex was at its minimum. With a fixed microchannel step width b_1_, increasing step height h_3_ led to an enlargement of the vortex area on the oxy plane. The vortex area size correlated directly with the flow rate at different cardiac cycle moments (Figure 4 and Figure 5).

**Figure 6:**
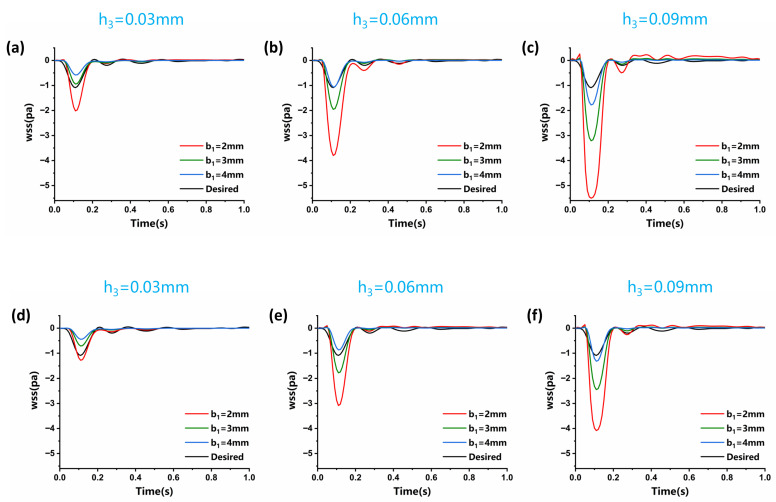
Evolution of the simulated low OWSS at the center point of the vortex at the observing point (y = −0.15 mm, z = 0 mm) under different conditions of step height and width for b_2_ = 5 mm (**a**–**c**) and b_2_ = 4 mm (**d**–**f**).

Correspondingly, within the vortex area, the negative oscillation amplitude of the WSS waveform decreased as the step width b_1_ increased (Figure 6). This decrease is attributed to reduced flow speed and subsequently lower velocity gradients under constant inlet flow rate conditions with increasing channel width. When keeping the step width b_1_ constant and altering step height h_3_, the size and position of the vortex area changed. As the step height h_3_ increased, the height and width of the vortex area increased, with its center shifting away from the step. The changes in the size of the vortex caused an increase in the negative amplitude of wall shear stress at the vortex center, accompanied by pronounced oscillations (Figure 6).

### 3.3. Influence of Wide Area Channel Size on Low Oscillatory WSS

**Figure 7:**
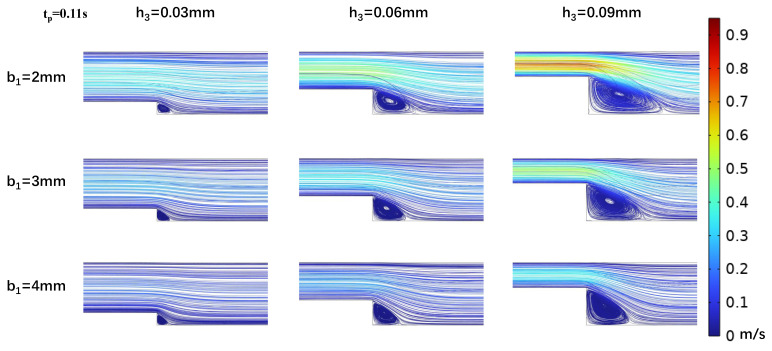
Changes in streamline distributions with step height and width at the stepped site in the time interval of the maximum flow velocity (t_p_ = 0.11 s) for b_2_ = 4 mm.

**Figure 8:**
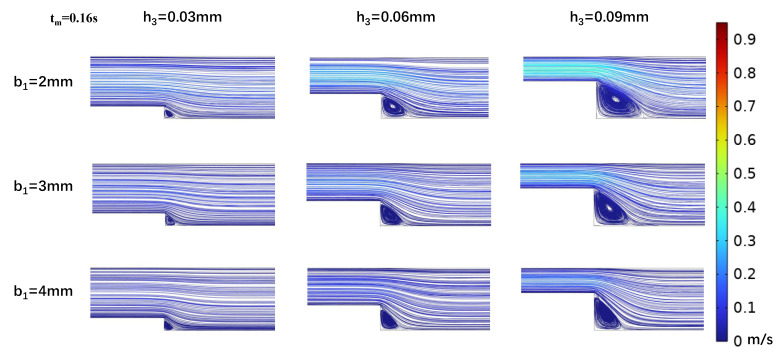
Changes in streamline distributions with step height and width at the stepped site in the time interval of the average flow velocity (t_m_ = 0.16 s) for b_2_ = 4 mm.

With a fixed target WSS for simulation in the wide area of the microchannel, the channel size in this area determines the internal flow rate (Equation (7)). As shown in Section 2.2, both vortex formation in the stepped region and the corresponding WSS variations during cardiac cycles exhibit strong dependence on the inlet flow rate. Therefore, we investigated the effect of flow rate variations on the vortices and WSS simulation at the step by altering the channel width b_2_. The simulations (Figure 7 and Figure 8) indicated that changing the channel width in the wide area b_2_ = 4 mm resulted in low OWSS at the step. The parametric dependence of vortex characteristics and OWSS distribution on step width (b_1_) and height (h_3_) exhibited identical trends to those observed at b_2_ = 5 mm (Figure 4, Figure 5, Figure 6, Figure 7 and Figure 8). Compared to b_2_ = 5 mm, the reduced flow rate in the microchannel at b_2_ = 4 mm diminished the vortex area (Figure 7 and Figure 8). Concurrently, the reduction in flow rate leads to a decrease in the negative oscillation amplitude of the WSS waveform at the center point of the vortex.

## 4. Discussion

In this study, we developed a variable cross-section microchannel with a stepped structure to replicate carotid sinus hemodynamics. CFD simulations confirmed that the optimized stepped structure accurately reproduces the vortex formation and low OWSS characteristic of carotid sinus stenosis, while simultaneously generating high-PWSS waveforms in the downstream uniform channel section (Figure 3a). The physiological flow vortex and WSSs with different features can be simultaneously reproduced with our designed microfluidic chamber. This work provides a platform for simulating physiological WSS waveforms in the carotid artery and offers a foundation for future research into the mechanobiological mechanisms of arterial endothelial dysfunction induced by abnormal hemodynamic factors, such as vortex and low OWSS [4,5,6,7].

Parallel-plate flow chamber systems [14,15,16] have served as the predominant in vitro model system for mechanobiological studies, enabling controlled simulation of fluid shear stress microenvironments for endothelial cell culture. Due to the low Reynolds number and laminar flow, the physiological WSS waveforms can be precisely replicated by regulating the flow rate of introduced fluids. In our microchannel, the downstream section features micron-scale dimensions, resulting in a Reynolds number (Re) << 1 that ensures fully developed laminar flow. Therefore, the high-PWSS waveforms in the uniform sections downstream of the carotid sinus can be precisely simulated. Additionally, the developed channel showed an advantage in reproducing a vortex and simulating the WSS features in the vortex (Figure 4, Figure 5, Figure 6, Figure 7 and Figure 8). It is well recognized that high PWSS plays a role in preserving vascular endothelial functions, while vortex flow and low oscillatory WSS are abnormal hemodynamic factors [4,5,6,7,8]. These normal and abnormal hemodynamic factors can be simultaneously replicated with our proposed microchannel. Therefore, the designed microfluidic chamber is suitable for comparing the differential impact of hemodynamic factors on vascular endothelial functions.

Our CFD simulation results revealed that the existence of a vortex and features (e.g., negative amplitude and waveform) of low OWSS were significantly impacted by the step geometry. A decrease in entrance flow rate *Q*(t), increase in step width b_1_, and decrease in step height h_3_ will reduce and even eliminate the vortex (Figure 4 and Figure 5). The variation in WSS is more sensitive to step height due to the inverse square relation (Equation (7)). Thus, it is necessary to optimize the geometry and flow conditions of the microfluidic chamber. Here, we have described the optimization procedure (Figure 2) and the optimization criterion. But the limitation is that the optimization process is manually adjusted step by step until the criterion is reached. The optimization should be improved in future studies. Moreover, due to the irregularity of the vortex, it is impossible to reproduce the whole region of a physiological existing vortex in vivo. Only the main features of the vortex and WSS can be captured and simulated in local parts within the microchannel. This limitation leads to a narrow area with homogenized WSS, which may limit the number of cultured cells for mechanobiological experiments.

Although this study successfully optimized and validated a microfluidic platform capable of replicating complex wall shear stress (WSS) waveforms found at the carotid sinus, several limitations should be acknowledged. Firstly, the optimized vortex region is spatially confined, which restricts the number of cells available for population-level biochemical assays. To address this, future strategies may involve designing parallelized multi-channel chips to increase cell yield [37]. Consequently, the current device may be particularly suitable for high-resolution imaging-based single-cell mechanobiology studies rather than population-level analyses. Secondly, the manual parameter-sweep optimization approach employed, while effective, is computationally expensive and not easily generalizable. Future work will explore automated optimization frameworks leveraging artificial intelligence, such as Bayesian optimization for fluidic systems [38], to enable efficient adaptation to complex pathological geometries.

Furthermore, experimental validation and biological application represent the essential next steps. The device will be fabricated in PDMS via soft lithography and integrated with a precision pulsatile pump system. WSS profiles will be experimentally validated using micro-particle image velocimetry [39]. Biological studies will utilize human endothelial cells to investigate spatial heterogeneity in inflammatory marker expression (e.g., ICAM-1, VCAM-1) and assess endothelial permeability under simultaneously applied high-positive and low-oscillatory WSS conditions—key processes in atherogenesis [40].

## 5. Conclusions

In this study, leveraging microfluidic technology and fluid dynamics principles, we developed a variable cross-section microchannel with a stepped structure as a cell culture chamber. With a step height of 0.06 mm, a width of 4 mm, and a wider microchannel width of 5 mm, the proposed microfluidic chamber is capable of simultaneously replicating both the low OWSS found at the carotid sinus and the high-PWSS waveform characteristics in the downstream uniform arterial segment as observed in vivo. Additionally, the formation of vortices and variations in low OWSS within the stepped region of the microchannel correlate with the step height, width, and dimensions of the wider channel area. An increase in step width b_1_ leads to a decrease in both the vortex area and the magnitude of negative WSS oscillations. When the step height h_3_ falls below 0.03 mm, the vortex area approaches to disappear, challenging the simulation of low oscillatory WSS patterns. The variable cross-section microchannel designed in this research lays a foundational basis for simulating carotid artery WSS waveforms and for conducting in vitro studies on the interplay between low OWSS at the site of carotid sinus stenosis and arterial endothelial dysfunction.

## Figures and Tables

**Table 1:** Definitions and units of main symbols.

Symbols	Expression	Unit
$b_{1}$	Part (I) width	mm
$h_{1}$	Part (I) height	mm
$L_{1}$	Part (I) length	mm
$b_{2}$	Part (II) width	mm
$h_{2}$	Part (II) height	mm
$L_{2}$	Part (II) length	mm
$h_{3}$	Step height	mm
$t_{p}$	Time of maximum velocity	s
$t_{m}$	Mean time of velocity	s
l	Distance from the step	mm
x_(1–5)_	5 observation points downstream from the step	mm
x_(6–10)_	5 observation points at part (II)	mm
$\vec{V}$	Fluid velocity	m/s
μ	Fluid viscosity	Pa · s
ρ	Liquid density	kg/m
$\vec{V_{i}}$	Inlet velocity	m/s^2^
*Q*(t)	Volume flow rate of the inlet fluid	m^3^/s
$\vec{V_{w}}$	Velocity at channel wall	m/s
$p_{0}$	Pressure at the outlet	Pa
$\tau_{w1}$(t)	WSS at the channel bottom of Part (I)	Pa
$\tau_{w2}$(t)	WSS at the channel bottom of Part (II)	Pa
$\vec{j}$	Unit vector in the y direction	

## Data Availability

The data that support the findings of this study are available from the corresponding author upon reasonable request.

## Supplementary Materials

The following supporting information can be downloaded at: https://www.mdpi.com/article/10.3390/bios15100648/s1, Figure S1: Waveform of flow rate at the common carotid artery measured by ultrasound Doppler; Figure S2: Microchannel meshing with different elements and the values of WSS at 24 mm and 14.025 mm, when t = 0.11 s.

## Abbreviations

The following abbreviations are used in this manuscript:

WSS	Wall shear stress
CFD	Computational fluid dynamics
OWSS	Oscillatory wall shear stress
PWSS	Pulsatile wall shear stress
3D	Three-dimensional
CCA	Common carotid artery
ICA	Internal carotid artery
ECA	External carotid artery
RMSE	Root mean square error
