# Supplementary material for: A Variable Cross-Section Microfluidic Channel for Simultaneous Reproduction of Low Oscillatory and Pulsatile Wall Shear Stress at the Carotid Bifurcation: A Computational Fluid Dynamics-Based Study
Source: Biosensors (Basel). 2025 Sep 30;15(10):648. doi: 10.3390/bios15100648 (PMC12564271; doi:10.3390/bios15100648)
Supplement: Supplementary file 1 [file biosensors-15-00648-s001.zip › biosensors-3787517-supplementary.pdf]

SUPPLEMENTARY FIGURE

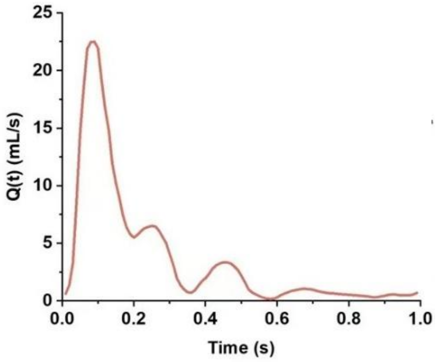

Figure S1. Waveform of flow rate at the common carotid artery measured by ultrasound Doppler.

Figure S2 shows the results of sensitivity to meshing elements number. The WSS corresponding to five levels of mesh densities (Ⅰ, Ⅱ, Ⅲ,Ⅳ, Ⅴ) were calculated. The results show that when the grid number reaches 2132789 , the WSS have reached convergence. Therefore, the meshing level Ⅳ is selected with the stepped area (x=13.5~14.5 mm) created using extra fine mesh and the other areas discretized with finer mesh to save computational resources.

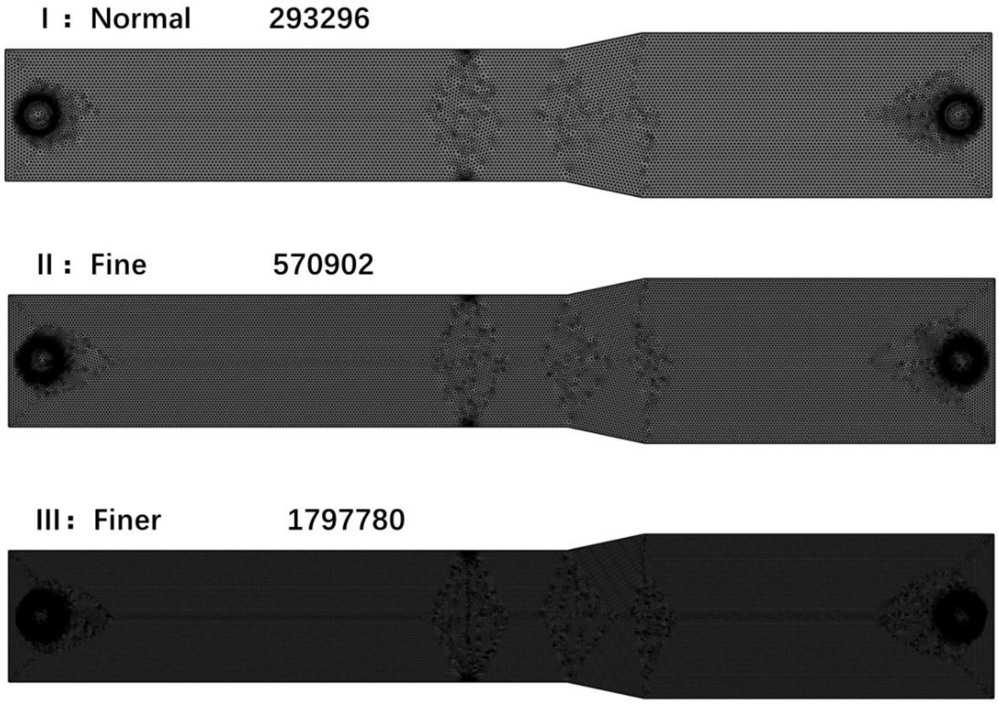

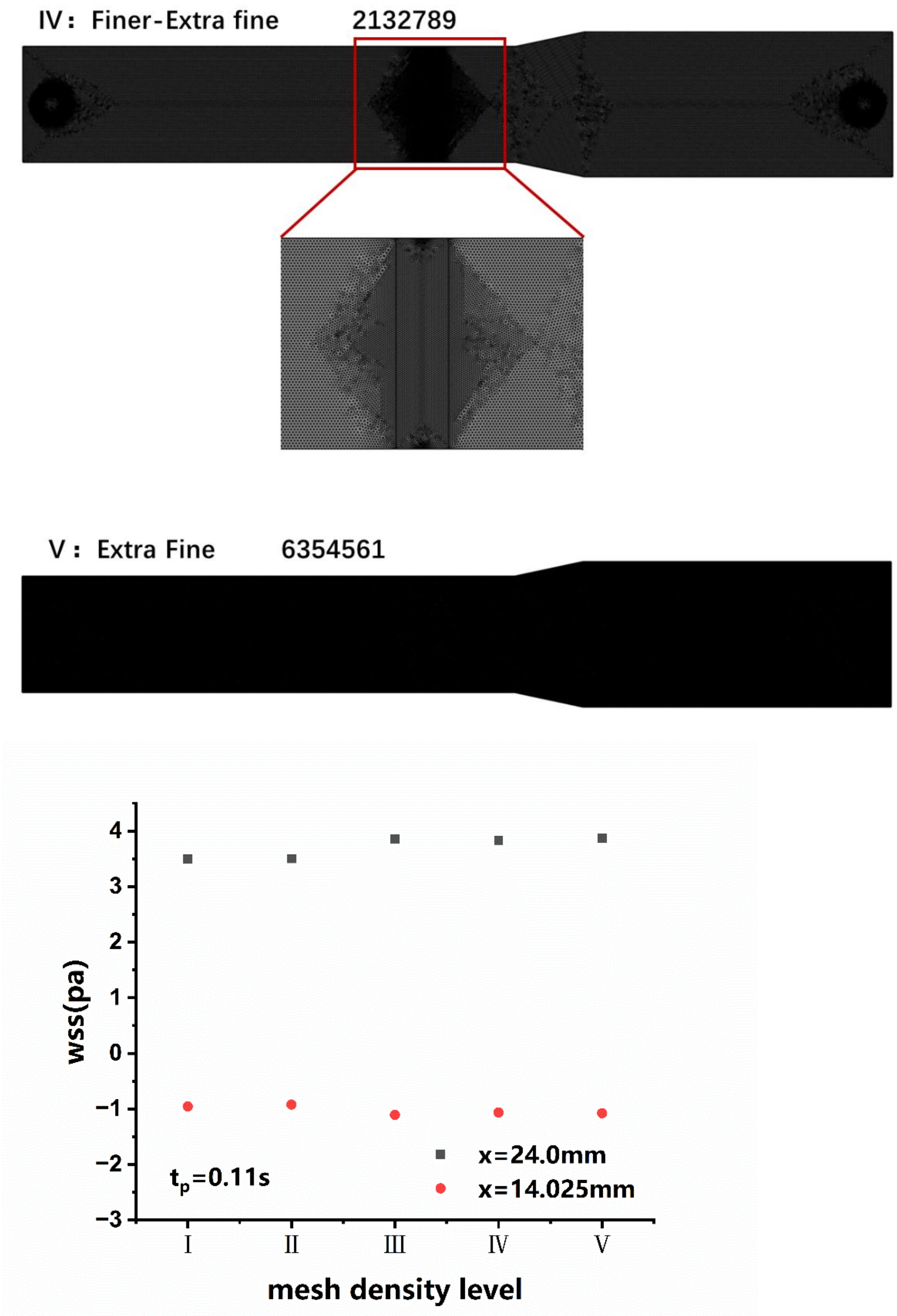

Figure S2. microchannel meshing with different elements and the values of WSS at 24mm and 14.025mm, when t = 0.11s. at 0.11s.
